# Advances in nanomedicine for cancer starvation therapy

**DOI:** 10.7150/thno.38261

**Published:** 2019-10-17

**Authors:** Shuangjiang Yu, Zhaowei Chen, Xuan Zeng, Xuesi Chen, Zhen Gu

**Affiliations:** 1 College of Material, Chemistry and Chemical Engineering, Hangzhou Normal University, Hangzhou 311121, P. R. China.; 2 Department of Bioengineering, Jonsson Comprehensive Cancer Center, California Nanosystems Institute (CNSI), and Center for Minimally Invasive Therapeutics, University of California, Los Angeles, CA 90095, USA.; 3 Key Laboratory of Polymer Ecomaterials, Changchun Institute of Applied Chemistry, Chinese Academy of Sciences, Changchun 130022, China. E-mail:

**Keywords:** drug delivery, nanomedicine, cancer starvation therapy, combination treatment

## Abstract

Abnormal cell metabolism with vigorous nutrition consumption is one of the major physiological characteristics of cancers. As such, the strategy of cancer starvation therapy through blocking the blood supply, depleting glucose/oxygen and other critical nutrients of tumors has been widely studied to be an attractive way for cancer treatment. However, several undesirable properties of these agents, such as low targeting efficacy, undesired systemic side effects, elevated tumor hypoxia, induced drug resistance, and increased tumor metastasis risk, limit their future applications. The recent development of starving-nanotherapeutics combined with other therapeutic methods displayed the promising potential for overcoming the above drawbacks. This review highlights the recent advances of nanotherapeutic-based cancer starvation therapy and discusses the challenges and future prospects of these anticancer strategies.

## 1. Introduction

Characterized by abnormal cell metabolism and growth with risk of metastasis, cancer remains a global fatal threat to human health today 1, 2. In recent years, cancer starvation therapy is emerging as an effective method for suppressing tumor growth and survival through blocking blood flow or depriving their essential nutrients/oxygen supply 3-5. The transport of nutrients could be blocked by stopping the tumor blood supply with the treatments of angiogenesis inhibiting agents (AIAs) 6, 7, vascular disrupting agents (VDAs) 8, 9 and transarterial chemoembolization (TACE) 10. Moreover, agents that could consume the intratumoral nutrients/oxygen or mediate the essential substances uptake by tumor cells can also lead to tumor “starvation” and necrosis 4, 5, 11, 12. Although some unique advantages have been exhibited for cancer treatment these years, concerns associated with these agents, such as low targeting efficiency, elevated tumor hypoxia, acute coronary syndromes, abnormal ventricular conduction, induced drug resistance and increased tumor metastasis risk, limit their further applications in clinic 13-16.

To overcome these challenges, combination therapy of cancer starvation agents with other cancer treating approaches has demonstrated to be an efficient way, which can maximize the therapeutic efficiency when compared to the single therapeutic method alone 17. However, issues of the free drugs, such as undesirable drug absorption, poor bioavailability and rapid metabolism* in vivo*, have still been concerned 18. The advances in micro-/nanotechnology as well as cancer biology have boosted development of drug delivery systems for cancer management with enhanced efficacy and limited side effects 19-22. Among them, a variety of nanomaterials based on natural/synthetic polymers 23-29, liposomes 30, metal-organic frameworks (MOFs) 13, gold nanoparticles (NPs) 31 and silica NPs 11, 32, 33 have been employed to co-deliver cancer-starving agents and other therapeutics with the aim of reducing drug side effects 23, improving their targeting efficacy 26, 27, increasing the stability and half-life of therapeutics 13, and co-delivery of multiple drugs to overcome the drug resistance 34, 35. Furthermore, cancer-starvation strategy associated with the multimodal nanomedicines have also been developed for achieving synergistic cancer therapy, which has been demonstrated to be the efficient way for overcoming the side effects of free drugs and resulting in superadditive therapeutic effects 14, 15, 20.

**Figure 1:**
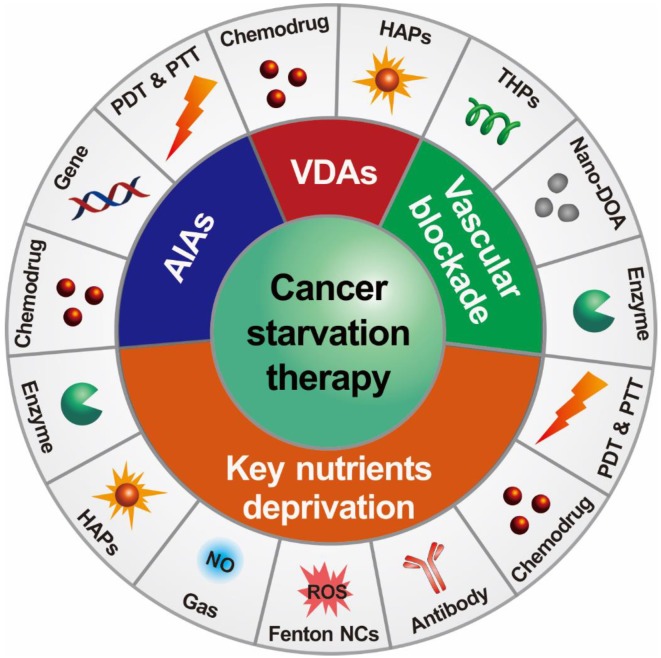
Schematic illustration of nanomedicine-mediated cancer starvation therapy. AIA: angiogenesis inhibiting agent; Nano-DOA: nano-deoxygenation agent; HAP: hypoxia-activated prodrug; NO: nitric oxide; NC: nanocatalyst; PDT: photodynamic therapy; PTT: photothermal therapy; ROS: reactive oxygen species; THP: tumor-homing peptide; VDA: vascular disrupting agent.

There are two major mechanisms in designing starving-nanotherapeutics. One is stopping/reducing the tumor blood supply through inhibiting/disrupting angiogenesis, or directly blocking the blood vessels 11, 23, 26, 36, 37. The other is depriving essential nutrients/oxygen input of tumor cells through consuming the intratumoral nutrients/oxygen, or limiting the critical nutrients uptake 4, 38-40. For maximizing the therapeutic efficiency, these therapeutics were cooperated with other cancer treating approaches, including chemotherapy 41, 42, gene therapy 43, phototherapy 44, 45, gas therapy 46, and immunotherapy 47. Herein, we overview the recent efforts of leveraging nanomedicine-based drug delivery systems for cancer starvation therapy and focus on the major strategies of multimodal synergistic starvation treatments (**Figure 1**). Both the design principles and their anticancer performance of these formulations are highlighted. Finally, the challenges and future prospects of this field are discussed.

## 2. Nanomedicine-mediated cancer starvation therapy

### 2.1 Antiangiogenesis-related cancer starvation therapy

Tumor growth and metastasis highly depend on the angiogenesis, which is an essential step of neoplasms from benign to malignant transformation 48. Anti-angiogenic therapy provides an efficient way for arresting the tumor growth through inhibiting the key angiogenic activators 7, 49. Several AIAs have been approved by the Food and Drug Administration (FDA) for clinical cancer treatment since 2003 7. However, associated toxicities of these AIAs are nonnegligible according to the clinical/preclinical investigation, which includes hypertension, vascular contraction, regression of blood vessels and proteinuria 14, 17, 50.

#### 2.1.1 Nano-antiangiogenesis-based cancer monotherapy

Compared to the free AIAs, nanomedicine could both improve their therapeutic outcomes *via* regulating their release behavior and increasing the drug accumulation in the tumor site through the enhanced permeability and retention (EPR) effect as well as actively targeting the tumor and/or endothelial cells *via* surface conjugation with target ligands 51, 52. For example, mesoporous silica nanoparticles (MSNs) could significantly improve the targeting efficacy of tanshinone IIA (an angiogenesis inhibitor) to HIF-1*α* overexpression, leading to improved antiangiogenesis activity in a mouse colon tumor model (HT-29) 53. Several over-expressed receptors, such as integrin *α*_v_*β*_3_ and Neuropilin-1, were employed as the targets of nanomedicines, which showed enhanced targeting efficacy and improved tumor inhibiting rate 54-56. Furthermore, paclitaxel (PTX) loaded antiangiogenic polyglutamic acid (PGA)-PTX-E-[c(RGDfK)_2_] nano-scaled conjugate could markedly suppressed the growth and proliferation of the *α*_v_*β*_3_-expressing endothelial cells (ECs) and several cancer cells 57. Additionally, bevacizumab, an angiogenesis inhibitor against vascular endothelial growth factor (VEGF) was directly used as a targeting ligand to modify magnetic iron oxide nanoparticles (IONPs), which was demonstrated to be an efficient platform for bevacizumab delivery in mice breast tumor (4T1) treatment 58.

Nanonization strategies for AIAs not only could reduce their associated toxicities and enhance the antitumor efficacy to some degree, but also provide a multidrug co-delivery platform toward enhancing the AIAs-based combination anticancer efficacy 31, 34, 59-61.

#### 2.1.2 Synergistic antiangiogenesis/chemotherapy

Angiogenesis inhibitors were often used together with chemotherapeutics for overcoming their shortages and enhancing the antitumor efficacy 17. Recently, types of engineered anti-angiogenic nanotherapeatics have been developed for cancer combination treatment. For instance, doxorubicin (DOX) and mitomycin C (MMC) co-loaded polymer-lipid hybrid nanoparticles could significantly increase the animal survival and tumor cure rate compared with liposomal DOX for treating multidrug resistant human mammary tumor xenografts 34. DOX combining with methotrexate (MTX), which was co-delivered by MSNs could also significantly improve the efficacy of oral squamous cell carcinoma treatment through down-regulating the expression of lymph dissemination factor (VEGF-C) 62. Zhu and coworkers synthesized a matrix metalloproteinase-2 (MMP-2)-responsive nanocarrier for the co-delivery of camptothecin (CPT) and sorafenib, which was demonstrated to be an efficient approach for colorectal cancer synergistic therapy 63. Curcumin (Cur), a potent antiangiogenesis agent, was co-loaded with DOX into pH-responsive poly(beta-amino ester) copolymer NPs for the 4T1 tumor treatment, which showed intensive anti-angiogenic and pro-apoptotic activities 64.

#### 2.1.3 Synergistic antiangiogenesis/gene therapy

The co-delivery of antiangiogenesis drugs and gene silencing agents is considered to be another efficient way for cancer starvation therapy 43, 65-67. For example, Lima and coworkers synthesized a chlorotoxin (CTX)-conjugated liposomes for anti-miR-21 oligonucleotides delivery, which promoted the efficiency of miR-21 silencing and enhanced the antitumor activity with less systemic immunogenicity 68. Liu *et al.* also found that the fusion suicide gene (yCDglyTK) could induce tumor cell apoptosis more effectively after co-delivering with VEGF siRNA by a calcium phosphate nanoparticles (CPNPs), where the density of capillary vessels was also observed to obviously decrease in the xenograft tissue of gastric carcinoma (SGC7901) 67. Furthermore, the poly-VEGF siRNA/thiolated-glycol chitosan nanocomplexes were employed to help overcome the resistant problem of bevacizumab by Kim and coworkers 65. The results indicated that the combination of these two VEGF inhibitors produced synergistic effects with decreased VEGF expression and drug resistance.

#### 2.1.4 Synergistic antiangiogenesis/phototherapy

**Figure 2:**
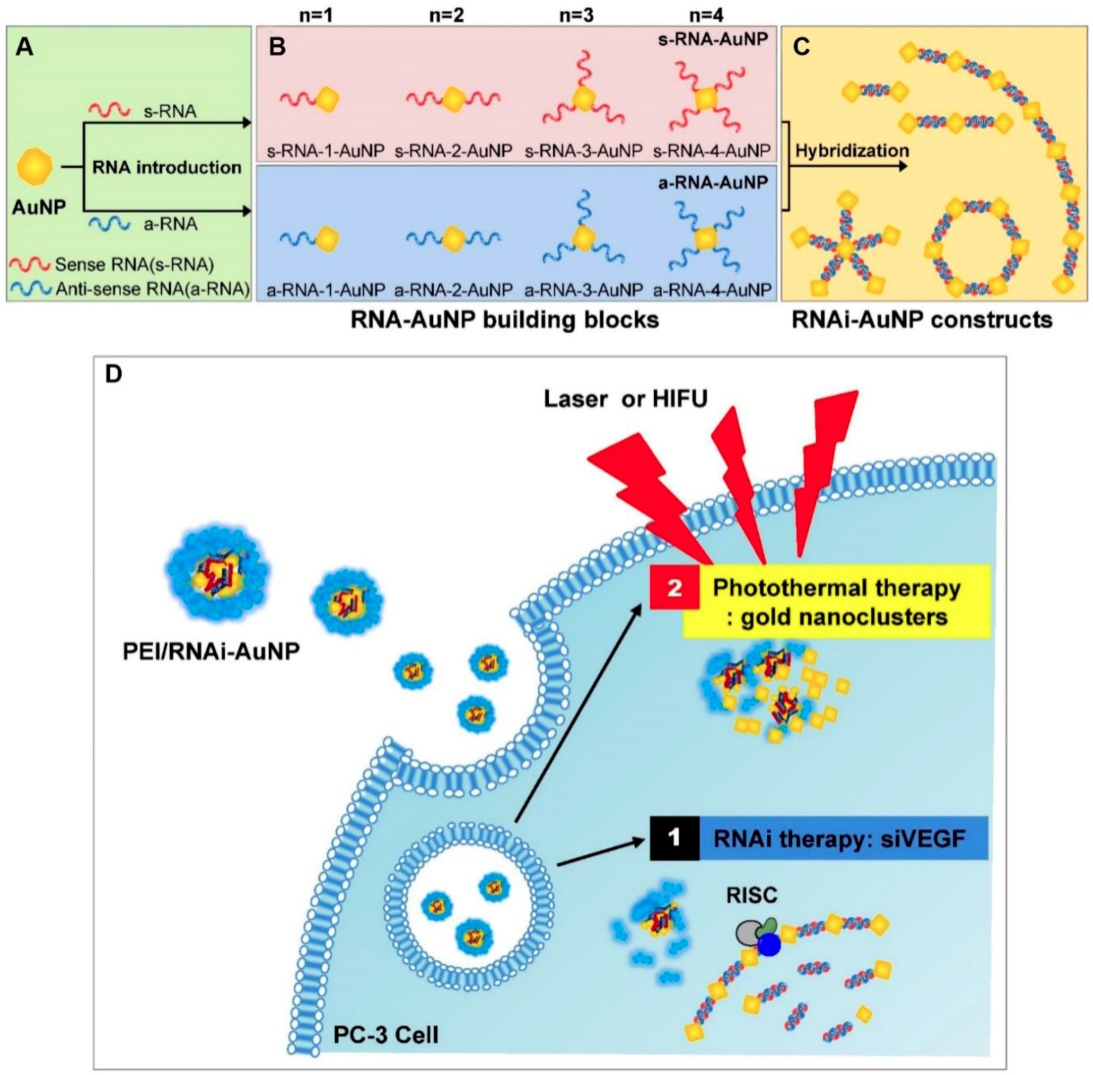
Schematic illustration of antisense- and sense-RNA strands introduction (A), RNA-AuNP building blocks with n-designated numbers of RNA strands (B) and versatile RNAi-AuNP nanoconstructs (C). Illustration of PEI/RNAi-AUNPs-induced the combinational strategy of anti-angiogenesis/photothermal cancer therapy (D). Reproduced with permission from ref. 31. Copyright 2017, Ivyspring international publisher.

Nanomaterial-based phototherapies that can selectively kill cancer cells without normal tissue injury have attracted extensive interest in the field of cancer treatments 69-71. Enhanced antitumor efficacy was also observed when angiogenesis inhibitors and phototherapy agents were combined 31, 72. For example, Kim and coworkers developed a hybrid RNAi-based AuNP nanoscale assembly (RNAi-AuNP) for combined antiangiogenesis gene therapy and photothermal ablation **(Figure 2)** 31. AuNPs modified by single sense/anti-sense RNA strands could self-assemble into various geometrical nanoconstructs (RNAi-AuNP). Then, PEI/RNAi-AuNP complexes were prepared with branched polyethylenimine (BPEI) for the purpose of effective intracellular delivery. After intratumoral administration, the therapeutic effects of PEI/RNAi-AuNP complexes could be activated by continuous-wavelength lasers or high intensity focused ultrasound, which led to effective antiangiogenesis and tumor ablation. In another work, a carrier-free nanodrug was prepared by self-assembling of Sorafenib and chlorin e6 (Ce6) for antiangiogenesis and photodynamic therapy 72. This nanodrug presented good passive targeting behavior in the tumor sites and effective reactive oxygen species (ROS) generation ability *in vivo*. The tumor inhibition rate was significantly improved after combination with Sorafenib. With additional merits, such as good biosafety and biocompatibility, this nano-integrated strategy promised potential for cancer synergetic treatment in clinic.

### 2.2 VDAs-based cancer starvation therapy

VDAs, as a unique class of anticancer compounds, is designed to selectively prevent the established abnormal tumor blood vasculature by targeting ECs and pericytes, leading to tumor starvation and central necrosis through hypoxia and nutrient deprivation 73. However, they are powerless to the cancer cells at the tumor margin, which could draw oxygen and nutrients from the surrounding normal tissues 15. Beside this, several other vascular risk factors, such as the acute coronary syndromes, blood pressure alteration, abnormal ventricular conduction, and transient flush, also limit the further application of free VDAs 73. To overcome the above issues and enhance their antitumor ability, VDAs-based multimodal cancer therapies have been developed for solid tumor treatments 23, 27, 28, 42, 74-78.

#### 2.2.1 Free VDAs-enhanced nanomedicine-based chemotherapy

The barriers of heterogeneity and high interstitial fluid pressure of solid tumors not only limit the targeting efficiency of nanomedicines, but also weaken their antitumor ability against the tumor central area 79, 80. Recent studies reported that small free molecule VDAs could help nanomedicines to overcome the above drawbacks 42, 74, 75. For example, Chen and coworkers developed a coadministration strategy using free CA4P and CDDP-loaded PLG-*g*-mPEG NPs (CDDP-NPs) for complementing each other's antitumor advantages and improving the antitumor efficiency 75. The multispectral optoacoustic tomography (MSOT) images indicated that the tumor penetration of CDDP-NPs highly relied on the tumor vasculature, which aggregated in the peripheral region of the tumors. While co-administration of free CA4P and CDDP-NPs improved the tumor cellular killing efficiency both in the central and peripheral regions according to hematoxylin and eosin (H&E) staining. The enhanced antitumor efficiency against both murine colon cancer (C26) and human breast cancer (MDA-MB-435) models supported that this combination strategy was a promising way for solid tumor treatment.

Furthermore, small molecule VDAs could induce tumor target amplification of ligand-coated NPs through selectively modifying tumor vasculature. For example, protein p32, a stress-related protein which is specifically expressed on the surface of tumor cells 37, can selectively bind with the phage-displayed cyclic peptide (LyP-1) 81. Ombrabulin, a small molecule VDAs, was used to induce the local upgraded presentation of protein p32 for enhancing the tumor “active targeting” of LyP-1 coated NPs. The *in vivo* results demonstrated that the recruitment of LyP-1 coated DOX-loaded NPs significantly increased after pretreating with ombrabulin when compared with the control groups 74. In another work, coagulation-targeted polypeptide-based NPs were developed for improving their tumor-targeting accumulation by homing to VDA-induced artificial coagulation environment. The* in vivo* results showed that this cooperative targeting system recruited over 7-fold higher CDDP doses to the tumors than non-cooperative control groups 42. The above cooperative targeting strategies combining with free VDAs and ligand-coated NPs showed obviously decreased tumor burden and prolonged mice survival compared to the non-cooperative controls.

#### 2.2.2 VDAs-nanomedicine induced synergistic starvation/chemotherapy

VDAs-nanomedicine could enhance their accumulation and retention at the leaky tumor vasculature *via* EPR effect, leading to high distribution and gradual release of VDAs around the immature tumor blood vessels as well as prolonged vascular disruption effect compared to free drugs 28. Beside this, nanomedicine also provides a platform for VDAs-based cancer multimodal therapy 23, 27, 76, 78. For instance, a multi-compartmental “nanocell” integrating a DOX-PLGA conjugate core and a phospholipid shell was prepared for achieving temporal release of DOX and combretastatin A4 (CA4) 23. After accumulating at the tumor site, CA4 was released from the outer phospholipid shell of the nanocell rapidly and attacked the tumor blood vessels, and DOX was then released subsequently from the inner polymeric core for killing tumor cells directly. This mechanism-based strategy exhibited reduced side toxicity and enhanced therapeutic synergism in the progress of inhibiting murine melanoma (B16F10) and Lewis lung carcinoma growth.

**Figure 3:**
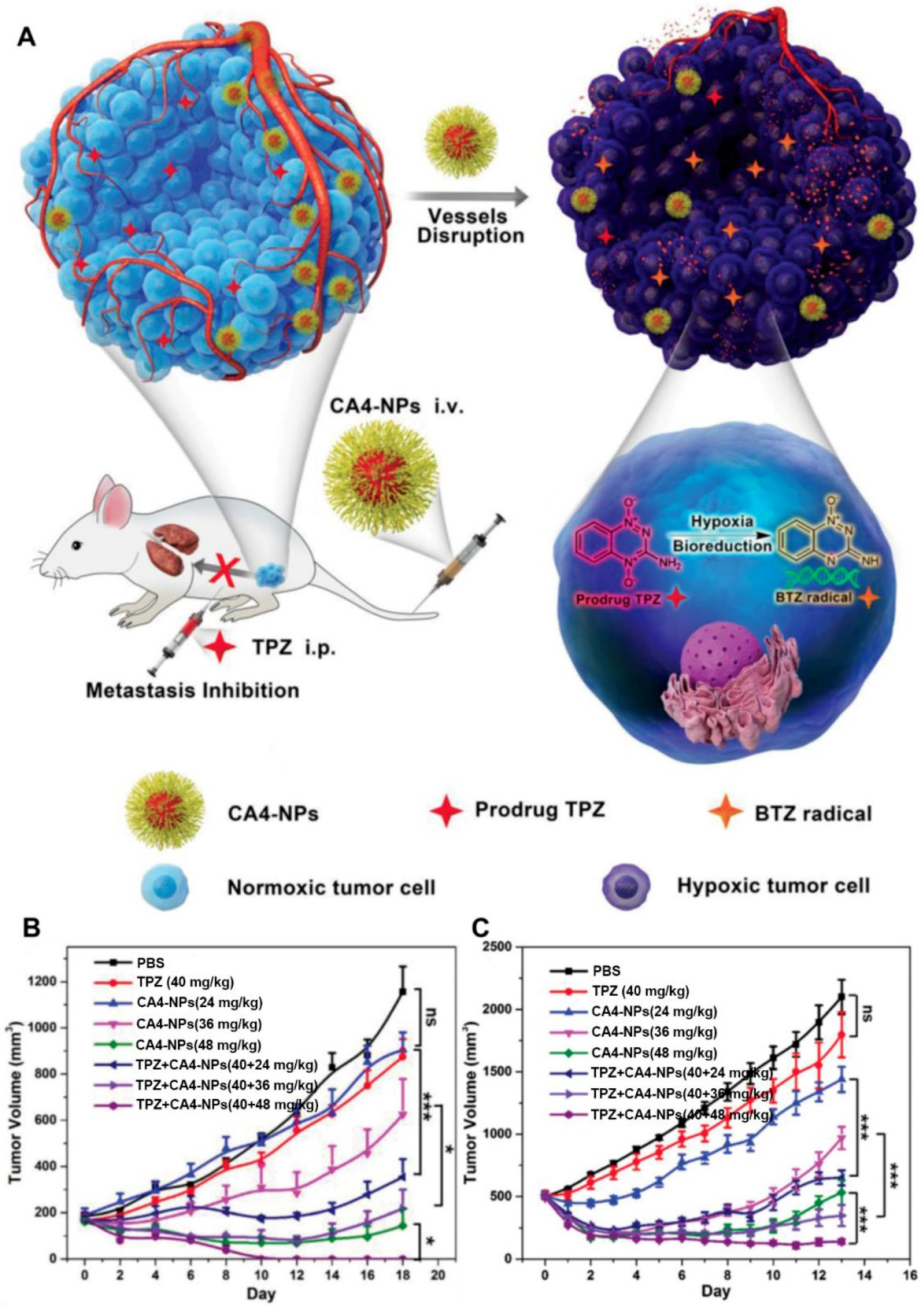
Schematic illustration of hypoxia-inducing VDA nanodrug combined with hypoxia-activated prodrug for cancer therapy (A). Tumor volume changes of BALB/c mice bearing 4T1 tumors with both moderate sizes (≈180 mm^3^) (n=6). (B). and large sizes (≈500 mm^3^) (n=6). (C). All data points are presented as mean ± standard deviation (s.d.). (*P<0.05, **P<0.01, ***P<0.001). Reproduced with permission from ref. 27. Copyright 2019, Wiley-VCH.

Furthermore, several polymer-VDA conjugates caused amplified TME characteristics was also utilized to develop new cancer co-administration strategies 27, 78. Hypoxia is one of the major features of solid tumors which can promote neovascularization, drug resistance, cell invasion and tumor metastasis 82, 83. Meanwhile, the existence of hypoxia also provides the desired target for tumor selective therapy 21. Tirapazamine (TPZ) is a typical hypoxia-activated prodrug (HAP), which own low toxicity toward normal tissues and can selectively kill the hypoxic cells after conversion into cytotoxic benzotriazinyl (BTZ) radical within hypoxic regions 84. Nevertheless, the insufficient hypoxia level within tumors tremendously limited its further clinical application 85. To address this, Chen and coworkers proposed a cooperative strategy based on VDA-nanomedicine and HAPs for solid tumor treatment** (Figure 3)** 27. In this study, poly(L-glutamic acid)-CA4 conjugate nanoparticles (CA4-NPs) were employed to selectively disrupt the abnormal vasculature of the tumor, as well as elevating the hypoxia level of the tumor microenvironment (TME). The intensive hypoxic TME further boosted the antitumor efficacy of TPZ subsequently. The *in vivo* results demonstrated that this combinational strategy can not only completely suppress the small tumor growth (initial tumor volume: 180 mm^3^), but also obviously keep down the size of large tumors (initial tumor volume: 500 mm^3^) without distal tumor metastasis. Moreover, Chen and coworkers also demonstrated that the expression of matrix metalloproteinase 9 (MMP9, a typical tumor-associated enzyme) in treated tumors (4T1) could be markedly increased by more than 5-fold after treatment with CA4-NPs. These overexpressed MMP9 could further activate the DOX release from a MMP9-sensitive doxorubicin prodrug (MMP9-DOX-NPs) and enhance the *in vivo* cooperative antitumor efficacy 78.

### 2.3 Vascular blockade-induced cancer starvation therapy

Besides the strategies of anti-angiogenic therapy and VDAs-induced tumor blood vessel disrupting, another promising strategy for cancer starvation therapy was proposed by shutting off the blood supply with nanothereapeutics that could selectively blockade tumor vascular and then inducing tumor necrosis.

#### 2.3.1 Tumor-homing peptides-induced cancer starvation therapy

Tumor-homing peptides (THPs), such as pentapeptide (CREKA) and 9-amino acid cyclic peptide (CLT-1), could specially bind with fibrin-fibronectin complex in tumor blood clots 86. Based on this, Ruoslahti and coworkers developed a CREKA modified IONPs for fibrin-fibronectin complexes targeting and subtle clotting in tumor vessels 87. The initial deposition of these CREKA-IONPs created new binding sites for the subsequent NPs, and further enhanced the blood coagulation in the tumor lesion. The results indicated that the tumor imaging efficiency of this self-amplifying tumor homing system owned about six-fold enhancement compared to the control groups. However, the tumor inhibition efficiency of this system showed no significant improvement due to the insufficient tumor vessel occlusion. To this end, a cooperative theranostic system containing CREKA-IONPs and CRKDKC-coated iron oxide nanoworms was further developed by the same research group for improving the clots binding efficacy. The results proved that this combination system led to 60~ 70% tumor blood blockades and obvious tumor size reduction* in vivo* 88.

#### 2.3.2 Thrombin-mediated cancer starvation therapy

Thrombin is a serine protease that catalyzes series of coagulation-related reactions and leads to rapid thrombus formation during the clotting process 48. If thrombin can be precisely delivered to the tumor site and lead to selective occlusion of tumor-associated vessels by inducing the local blood coagulation, it might be a promising way for inhibiting the growth and metastasis of tumors. Recently, a nucleolin-targeting multifunctional DNA nanorobotic system was constructed for smart drug delivery. The presence of the nucleolin subsequently triggered the opening of these DNA nanotubes and released the loaded therapeutic thrombin, which then led to specific intravascular thrombosis and tumor vessel blockade at the tumor site 26. The growth of several tumor models was suppressed efficiently after treating with this thrombin-loaded DNA nanorobot, demonstrating that this system could become an attractive platform for cancer starvation therapy in a precise manner.

#### 2.3.3 Deoxygenation agent-induced cancer starvation therapy

It is known that insufficient oxygen (O_2_) supply could result in hypoxia-induced tumor cell necrosis 89. Based on this, Zhang *et al.* designed an injectable polyvinyl pyrrolidone (PVP)-modified magnesium silicide (Mg_2_Si) nanoparticle as a nano-deoxygenation agent (nano-DOA) for directly consuming the intratumoral O_2_ and starving tumors 11. This polymer-coated Mg_2_Si NPs could respond to the slightly acidic TME after the intratumoral injection, and be converted into silicon dioxide (SiO_2_) by scavenging the surrounding O_2_ at the tumor site. As a byproduct, the *in situ* formed SiO_2_ aggregates further occluded the tumor capillaries and obstructed the follow-up nutrient and O_2_ supply.

On the other hand, the intratumoral hypoxic level was also enhanced in the progress of O_2_ consuming with the presence of DOA. Given this reason, Bu and coworkers prepared a TPZ loaded PVP-modified Mg_2_Si nanoparticles (TPZ-MNPs) for drug delivery and combination cancer therapy 38. After intratumoral injection, the TPZ-MNPs quickly scavenged the O_2_ *in situ* and created an artificial anaerobic environment which caused the surrounding cell dormancy. Meanwhile, the released TPZ was activated in this promoted hypoxia TME, which further caused the now-dormant tumor cells death.

### 2.4 GOx-mediated cancer starvation therapy

Glucose is the major energy supplier for tumor growth and proliferation 90. Glucose oxidase (GOx) can specifically catalyze the conversion of glucose into gluconic acid and hydrogen peroxide (H_2_O_2_) with the involvement of O_2_. This reaction can directly consume glucose and O_2_, and elevate the local acidity, hypoxia and oxidation stress* in vivo*. Given this background, GOx has aroused considerable interest for cancer diagnosis and treatment in the past decade 4, 91. Nevertheless, there are several limitations of this approach when using GOx as an anticancer agent. On the one hand, the overproduced H_2_O_2_ of glucose oxidation can cause systemic toxicity and lethal chain reactions through directly damaging cell membranes, proteins and DNA of normal cells 92, 93. On the other hand, similar glucose supply and physiological requirement of normal cells often lead to off-targeting and ineffective starvation treatment 94. Through nanomedicine, GOx can co-delivery with other therapeutic agents for cancer multimodal treatments 4. Herein, we overview the recent representative GOx-based nanomedicines for cancer starvation therapy.

#### 2.4.1 GOx-based cancer monotherapy

GOx could be used as an antitumor agent alone through consuming the intratumoral glucose and making the tumor “starving”. The continuously generated H_2_O_2_ could further lead to DNA damage and tumor cell apoptosis 95, 96. For example, Dinda *et al.* prepared a GOx-entrapped biotinylated vesicle for active targeting cancer starvation therapy 97. This GOx-containing system showed about six-fold higher tumor cell killing efficiency compared to normal cells through depleting the glucose supply for tumor cells *in vitro*. However, the glucose depletion efficiency was restrained by the hypoxic TME *in vivo*, because of the insufficient O_2_ supply in the solid tumor. Therefore, a hyaluronic acid (HA)-coated GOx and MnO_2_ coloaded nanosystem (GOx-MnO_2_@HA) was constructed for enhancing cancer starvation therapy outcome 98. After uptaking by the CD44-expressing tumor cells, the local glucose was converted into gluconic acid and H_2_O_2_ with GOx catalysis. The generated H_2_O_2_ then reacted with MnO_2_ to generate O_2_, which further accelerated the local glucose-consumption. This nanosystem provided benefit to break the hypoxia obstacles and enhance the antitumor effect by GOx.

#### 2.4.2 Synergistic starvation/chemotherapy

**Figure 4:**
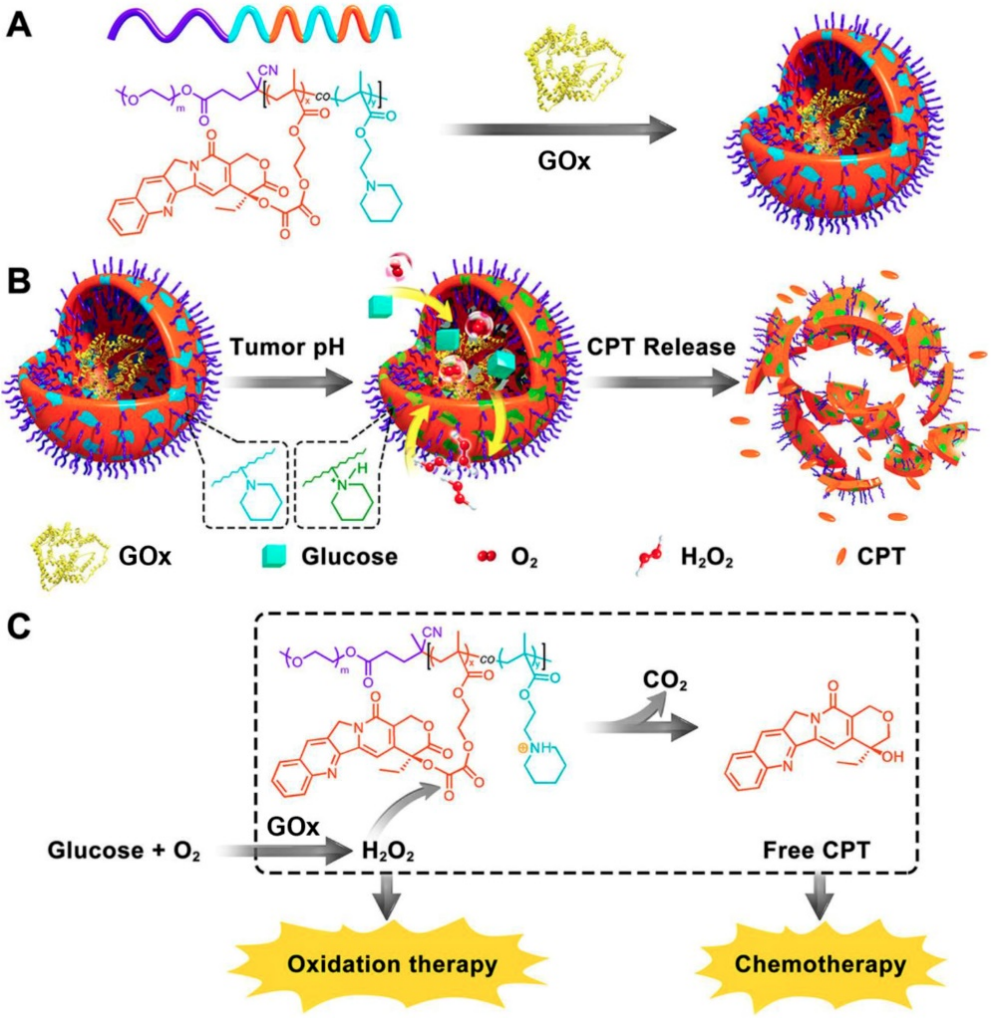
Schematic illustration of the preparation of GOx@PCPT-NR (A). Scheme of cancer starvation/chemotherapy *via in situ* H_2_O_2_ generation and acidity-activated CPT release (B). Molecular mechanism of GOx@PCPT-NR-induced cancer starvation/chemotherapy (C). Reproduced with permission from ref. 41. Copyright 2017, American Chemical Society.

As discussed above, the concentration of generated H_2_O_2_ can be substantially elevated in the presence of GOx at the lesion site. The increased H_2_O_2_ level was exquisitely used to active the H_2_O_2_-sensitive prodrugs for enhancing the synergistic efficiency of both cancer starvation and chemotherapy 41, 99. For example, Li *et al.* prepared a pH-responsive prodrug-based polymersome nanoreactor (GOx@PCPT-NR) that consisted of piperidine group, camptothecin (CPT) prodrug, PEG and GOx for cancer combination therapy **(Figure 4)** 41. This polymersome nanoreactor owned prolonged blood circulation and high tumor accumulation efficiency. The terminal elimination half-life of GOx@PCPT-NR reached above 39 hours after intravenous injection. This drug delivery system also showed excellent stability and almost no H_2_O_2_ and free CPT were found within 48 h treatment in the plasma or liver. Nevertheless, the slight acidity of the tumor (pH = 6.8) could trigger the GOx release from the polymersome, which then catalyzed the conversion of intratumoral glucose into gluconic acid and H_2_O_2_. The enhanced acidity causing by the generated gluconic acid could further promote the GOx release, while the elevated H_2_O_2_ level could further accelerate the active CPT release. The accumulating effects amplified the combination antitumor efficiency 41. Furthermore, this strategy was further confirmed by another biomimetic cascade nanoreactor (Mem@GOx@ZIF-8@BDOX) *in vivo*. As a byproduct of GOx-induced glucose depletion, gluconic acid could promote the release of loaded BDOX prodrug from the nano-framework, and the released BDOX were then converted into DOX in the presence of elevated H_2_O_2_ at the tumor site 99.

#### 2.4.3 GOx-inducing cancer starvation and hypoxia-activated chemotherapy

**Figure 5:**
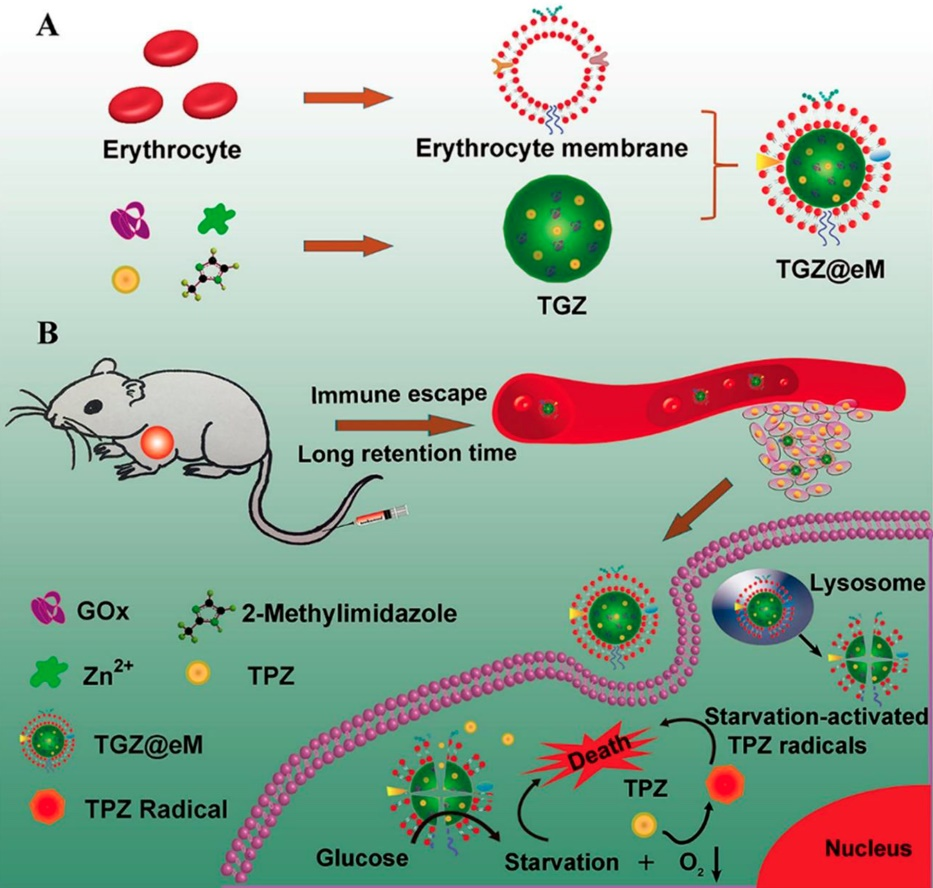
Schematic illustration of preparation of GOx and TPZ coloaded biomimetic nanoreactor with erythrocyte membrane coating (TGZ@eM) (A). TGZ@eM nanoreactor induced cancer starvation/hypoxia-activated chemotherapy (B). Reproduced with permission from ref. 13. Copyright 2018, American Chemical Society.

The consummation of molecular oxygen could increase the local hypoxia level in the progress of GOx-involved cancer therapy. This promoted hypoxic microenvironment was also employed to activate the hypoxia-activated prodrugs and amplify their antitumor activity 13, 30, 33, 100. For example, a MOF-based biomimetic nanoreactor coating with erythrocyte membrane (eM) was developed for precise GOx and TPZ delivery and cancer combination therapy **(Figure 5)** 13. The grafted biomimetic surface of the nanoreactor not only endowed it with prolonged blood circulation and immune-escaping property, but also enhanced the tumor homing efficiency of this nanosystem. After uptake by cells, the released GOx deprived the endogenous glucose and O_2_, which resulted in amplified hypoxic microenvironment and sufficient activation of TPZ. Based on the above synergistic cascade effects, a colon cancer model were efficiently inhibited *in vivo*. In another work, the PEG-modified long-circulating liposomes were used to sequentially deliver GOx and banoxantrone dihydrochloride (AQ4N, a hypoxia-activated prodrug) to tumors for cancer combination starvation/chemotherapy 30. The* in vivo* photoacoustic image indicated that GOx-loaded liposome could obviously deplete the glucose of the tumor site, and lead to tumorous hypoxia enhancement. Under the elevated hypoxic microenvironment, the antitumor activity of the subsequent arrival liposome-AQ4N was activated by reducing low toxic AQ4N into high toxic 1,4-Bis[[2-(dimethylamino)ethyl]amino]-5,8-dihydroxyanthracene-9,10-dione (AQ4) by the series of intracellular reductases. Synergistically enhanced antitumor effect was observed on 4T1 murine breast cancer model after treating with this liposome-based GOx/AQ4N co-delivery system. These results demonstrated that combination of GOx-based cancer starvation therapy and HAP-involved hypoxia-activated chemotherapy is an effective way for solid tumor treatment.

Furthermore, in order to reduce the systemic toxicity, Wang and coworkers developed a nanoclustered cascaded enzymes by crosslinking GOx and CAT with a pH-responsive block polymer poly(ethylene glycol)-*block*-poly(2-hydroxyethyl methacrylate) bearing 2-(2-carboxyethyl)-3-methylmaleic anhydride (PEG-*b*-PHEMA_CMA_) with a BSA/BSA_TPZ_ (wt:wt, 1:2) outer shell for cancer starvation and hypoxia-activated chemotherapy 94. The experimental data indicated that GOx and CAT could be released by the stimuli of the mild acidic TME after accumulating at the tumor site. Then, the release rate was self-accelerated by the subsequent generated gluconic acid with GOx-induced glucose consumption. Meanwhile, the aggravated hypoxia of TME further activated the BSA_TPZ_ which led to hypoxia-activated chemotherapy. Importantly, the authors also found that the present CAT could timely eliminate the appeared H_2_O_2_ as well as lowered the systemic toxicity of GOx-mediated cancer starvation therapy.

#### 2.4.4 Starvation/oxidation synergistic therapy

Glutathione (GSH) is a natural antioxidant in the body, which prevents the damage of important cellular components by ROS, such as H_2_O_2_, hydroxyl radicals (∙OH), and singlet oxygen (^1^O_2_). However, GSH could weaken the antitumor efficiency in the progress of ROS-mediated cancer therapy. To this end, Li *et al.* prepared GOx-loaded therapeutic vesicles based on a diblock copolymer containing a mPEG segment and copolymerized piperidine-functionalized methacrylate and phenylboronic ester (mPEG-*b*-P(PBEM-*co*-PEM)) 101. After precise activation at the tumor site, the GOx-induced enzymatic reaction caused local consumption of glucose and O_2_ and generation of gluconic acid and H_2_O_2_. The generated H_2_O_2_ not only elevated the intracellular oxidative stress, but also led to the production of quinone methide (QM), which further suppressed the antioxidant ability of the tumor cells through depleting the intracellular GSH. These cumulative anticancer effects of the therapeutic vesicles resulted in effective cancer cell death and tumor ablation.

H_2_O_2_ can be transformed into the highly toxic ROS under certain conditions *in vivo* 71, 102. For example, H_2_O_2_ could be disproportionated into ∙OH with the presence of Fenton reaction catalysts under acidic condition 103, 104. While, in the presence of neutrophil-expressed phagocytic enzyme myeloperoxidase (MPO), H_2_O_2_ and chlorine ion (Cl^-^) could be converted into hypochlorous acid (HClO) through the enzymatic reaction 105. Based on this, specific strategies were developed to combine with GOx for cancer starvation/oxidation synergistic therapy 106-110.

**Figure 6:**
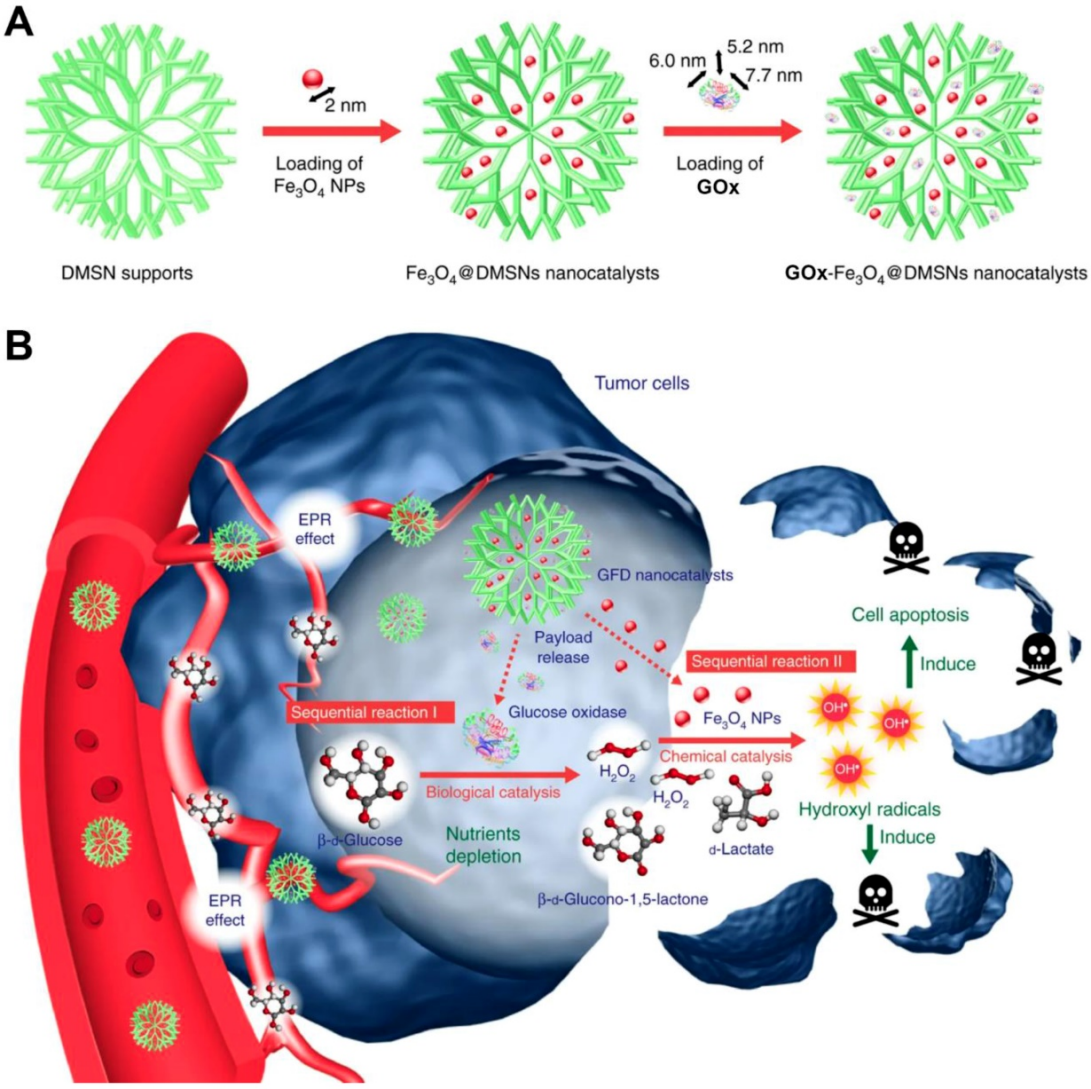
Synthetic procedure for preparation of GOx-Fe_3_O_4_@DMSNs (A). Scheme of GOx-Fe_3_O_4_@DMSNs induced sequential catalytic-therapeutic mechanism for cancer therapy (B). Reproduced with permission from ref. 106. Copyright 2017, Nature Publishing Group.

In a recent study, Huo *et al.* designed a dendritic silica nanoparticle-based sequential nanocatalyst for co-delivering of GOx and Fe_3_O_4_ NPs (GOx-Fe_3_O_4_@DMSNs) for enhancing of combination anticancer efficiency **(Figure 6)** 106. After EPR effect-induced accumulation of these nanocatalysts in the tumor site, the released GOx catalyzed the oxidation of the intratumoral glucose to gluconic acid and H_2_O_2_ and led to tumor starvation and central necrosis. Thereafter, the generated H_2_O_2_ sequentially were translated into highly toxic ∙OH by Fe_3_O_4_ NPs under the slightly acidic TME, which resulted in elevated oxidative stress and massive apoptosis of tumor cells. The final tumor inhibition rate of this nanomedicine by intravenous and intratumoral treatments with the same treating dose was reached to 64.7% and 68.9%, respectively. Besides this, another core-shell TME-responsive nanocatalyst, incorporated with a magnetic nanoparticle core of iron carbide (Fe_5_C_2_)-GOx and MnO_2_-nanoshell, was constructed by Lin and co-workers 107. After endocytosis by tumor cells, the MnO_2_-nanoshell of this nanosystem was degraded into Mn^2+^ and O_2_ and resulted in GOx release under the stimuli of the acidic microenvironment. The generated O_2_ could enhance consumption of the local glucose with the presence of GOx, leading to sufficient tumor starving. Sequentially, the produced H_2_O_2_ further evolved into ∙OH catalyzed by Fe_5_C_2_, resulting in efficient tumor cell death. Recently, a smart autocatalytic Fenton nanosystem, consisted of GOx-loaded zeolitic imidazolate framework (ZIF) and adenosine triphosphate (ATP)-responsive metal polyphenol network (MPN) shell, was designed by Zhang *et al*. for the combination cancer therapy 108. In tumor cells, the MPN shell was degraded into Fe^3+^ and tannic acid (TA) and further trigged the inner GOx release under the stimuli of the overexpressed ATP. Then, the exposed GOx led to endogenous glucose consumption and H_2_O_2_ accumulation. With the presence of TA, the transition efficiency of Fe^3+^ to Fe^2+^ was accelerated, which further promoted the transformation of the generated H_2_O_2_ into high toxic ∙OH by Fenton reaction. These accumulating antitumor effects significantly suppressed the tumor growth.

Silver (Ag) ions have been demonstrated to kill different types of cancer cells through increasing the intracellular oxidative stress, causing mitochondrial damage, and inducing cell autophagy 111, 112. Based on this, Huang and coworkers designed a GOx-conjugated silver nanocube (AgNC-GOx) for efficient Ag ions delivery and synergistic starvation/metal-ion therapy 113. AgNC-GOx catalyzed the glucose conversion into gluconic acid and H_2_O_2_ after uptake by the tumor cells. Cumulative gluconic acid elevated the acidity of TME which accelerated the AgNC degradation and Ag ions generation in the tumor site. Meanwhile, the generated H_2_O_2_ and Ag ions were found to lead the eradication of 4T1 cancer cells. Both the glucose consumption and accumulation of toxic H_2_O_2_ and Ag ions significantly suppressed the tumor growth and prolonged the mice survival.

**Figure 7:**
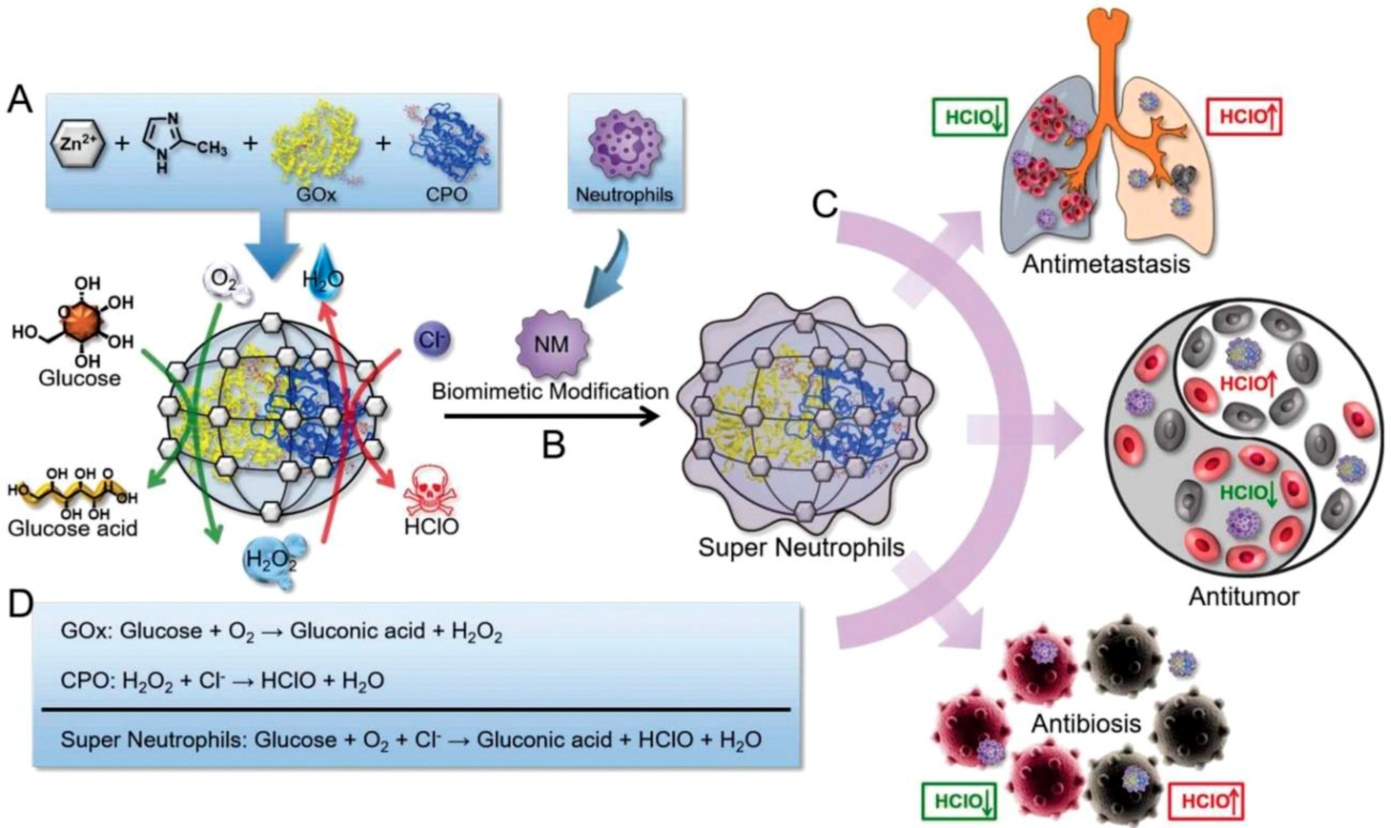
Schematic representation of the synthesis of GOx and CPO-coloaded ZIF-8 NPs. Glucose conversation and HClO generation with the catalyzing of GOx and CPO (A). Preparation of the “artificial neutrophils” by the surface modification of the GOx/CPO-coloaded ZIF-8 NPs with natural neutrophil membrane (B). Compare to natural neutrophils, the “artificial neutrophils” showed stronger HClO generation ability for biomedical applications (C). The mechanism of enzymatic reaction of GOx, CPO and “artificial neutrophils” (D). Reproduced with permission from ref. 109. Copyright 2019, Wiley-VCH.

HClO is a powerful ROS which can be generated by the MPO-mediated catalysis and owns higher cellular toxicity in comparison with H_2_O_2_. It has been proved to be a promising candidate for cancer therapy through disrupting some cellular functions and promoting the tumor cell death by the oxidation progress 114. Given this pattern, Zhang and coworkers prepared an “artificial neutrophils”, consisting of GOx and chloroperoxidase (CPO) coloaded zeolitic imidazolate framework-8 (ZIF-8) core and neutrophil membrane (NM) coating (GOx-CPO@ZIF-8@NM), for both of cancer and infection treatments. NM coating help the NPs to target the tumor site efficiently **(Figure 7)** 109. After uptake by the tumor cells, the embedded GOx and CPO were released from the ZIF-8 NPs, which synergistically enhanced the glucose depletion and HClO generation through a sequential enzymatic catalysis progress. According to the results, this artificial neutrophil can produced seven-fold higher reactive HClO than the natural neutrophils both *in vitro* and *in vivo*. Benefit from this, 4T1 tumors of mice was almost completely eradicated after treating with this neutrophil-mimicking NPs.

#### 2.4.5 Synergistic starvation/phototherapy

Blue light irradiation (450~490 nm) could promote the Conversion of H_2_O_2_ into more toxic ∙OH, which provides an alternative approach for cancer therapy. However, insufficient H_2_O_2_ supplies in the tumor site weaken the ∙OH production as well as the antitumor efficiency. Instead, GOx, as an antitumor agent, could induce the glucose depletion and tumor starvation with consecutively generating of H_2_O_2_. Given this fact, Chang *et al.* developed GOx-conjugated polymer dots (Pdot-GOx) for enzyme-enhanced phototherapy (EEPT) 115. After immobilizing into tumor, Pdot-GOx NPs could efficiently catalyze the glucose oxidation and steadily produce H_2_O_2_, which led to the enhancement of the local oxidative stress. Meanwhile, the appeared H_2_O_2_ could also be photolyzed to produce ∙OH under light irradiation (460 nm) for killing tumor cells. The experimental results indicated that this EEPT strategy exhibited much higher efficacy in inhibiting MCF-7 tumor growth compared with the control groups in mouse models.

Photodynamic therapy (PDT) has proved to be a promising platform in imaging and treatment of cancers and other diseases 116. As a noninvasive method, PDT utilizes the generated toxic ROS to destroy the cellular organelles and ablate tumors with the presence of photosensitizers under light irradiation. However, the poor penetration of the excitation light makes it powerless against deeply seated tumors 117. Besides, hypoxia of TME is another suppressive factor for this O_2_-dependent antitumor approach 118. Thus, the combination with other strategies would be an alternative way for improving the efficiency of PDT-involved cancer therapy 119, 120. Recently, Li *et al*. developed a GOx and catalase co-loaded porphyrin metal-organic framework with tumor cell membrane surface coating (mCGP) for synergistic starvation/PDT therapy. This mCGP NPs owned excellent tumor homing ability due to the tumor cell membrane surface coating. After internalized by tumor cells, the loaded catalase in mCGP was found to catalyze the generated H_2_O_2_ to disproportionate into molecular O_2_ and H_2_O, accelerating the consumption of endogenous glucose and promoting the production of ^1^O_2_ under light irradiation. These accumulating effects obviously enhanced the *in vivo* synergistic antitumor efficiency of mCGP NPs. In another work, Yu *et al*. developed a biomimetic nanoreactor (bioNR)-based starvation/PDT strategy for effective combating deeply seated metastatic tumors 32. The bioNR was constructed based on a GOx and Ce6 conjugated hollow mesoporous silica NPs (HMSNs) with B16F10 cell membrane coating, which was filled with bis[2,4,5-trichloro-6-(pentyloxycarbonyl)phenyl]oxalate (CPPO) and perfluorohexane (PFC) in the cavity. After homing to the tumor, the peripheral glucose was converted into gluconic acid with H_2_O_2_. At the same time, the appeared H_2_O_2_ not only promoted the local oxidative stress of the tumor, but also could react with CPPO to generate chemical energy, which led to chemiluminescence resonance energy transfer-based PDT with the presence of Ce6. Furthermore, the molecular oxygen releases from PFC further increase the antitumor efficiency of O_2_-dependent GOx-involved cancer starvation therapy and PDT. It was demonstrated that the Ce6-induced PDT effect for tumor metastasis was substantially enhanced after combination with GOx-involved cancer starvation therapy.

As described above, insufficient oxygen supply in solid tumors could limit the antitumor efficiency of GOx-related therapeutics. To address this challenge, Cai and coauthors prepared a HA-conjugated porous hollow Prussian Blue NPs (PHPBNs) for facilitating GOx delivery and tumor synergistic starvation/photothermal therapy 121. The HA shell could enhance the targeting efficiency towards CD44 overexpressing tumors. After cellular endocytosis, the released GOx catalyzed the glucose depletion by consuming O_2_, and PHPBNs sequentially catalyzed the generated H_2_O_2_ splitting into O_2_ and H_2_O to amplify the tumor starvation effect. Furthermore, GOx-induced glucose depletion not only inhibited the tumor growth, but also suppressed the expression of heat shock proteins (HSPs), where the latter facilitated PHPBNs-mediated low-temperature photothermal treatment to reduce their resistance. The results indicated that this combinational therapeutic system could significantly repress tumor growth in mice. In another work, Tang *et al*. developed a novel BSA-directed two-dimensional (2D) MnO_2_ nanosheet (M-NS) by one-step method 122. This M-NS not only owned an excellent GOx-like activity for catalyzing the local glucose oxidation, but also exhibited high photothermal conversion efficiency due to the large surface area. Furthermore, this M-NS artificial enzyme showed higher thermal stability than natural GOx. The experimental results indicated that the M-NS-induced intratumoral glucose depletion inhibited the ATP production as well as cellular HSPs expression, which promoted the sensitivity of tumors to the M-NS-mediated photothermal treatments.

#### 2.4.6 Starvation/gas synergistic therapy

**Figure 8:**
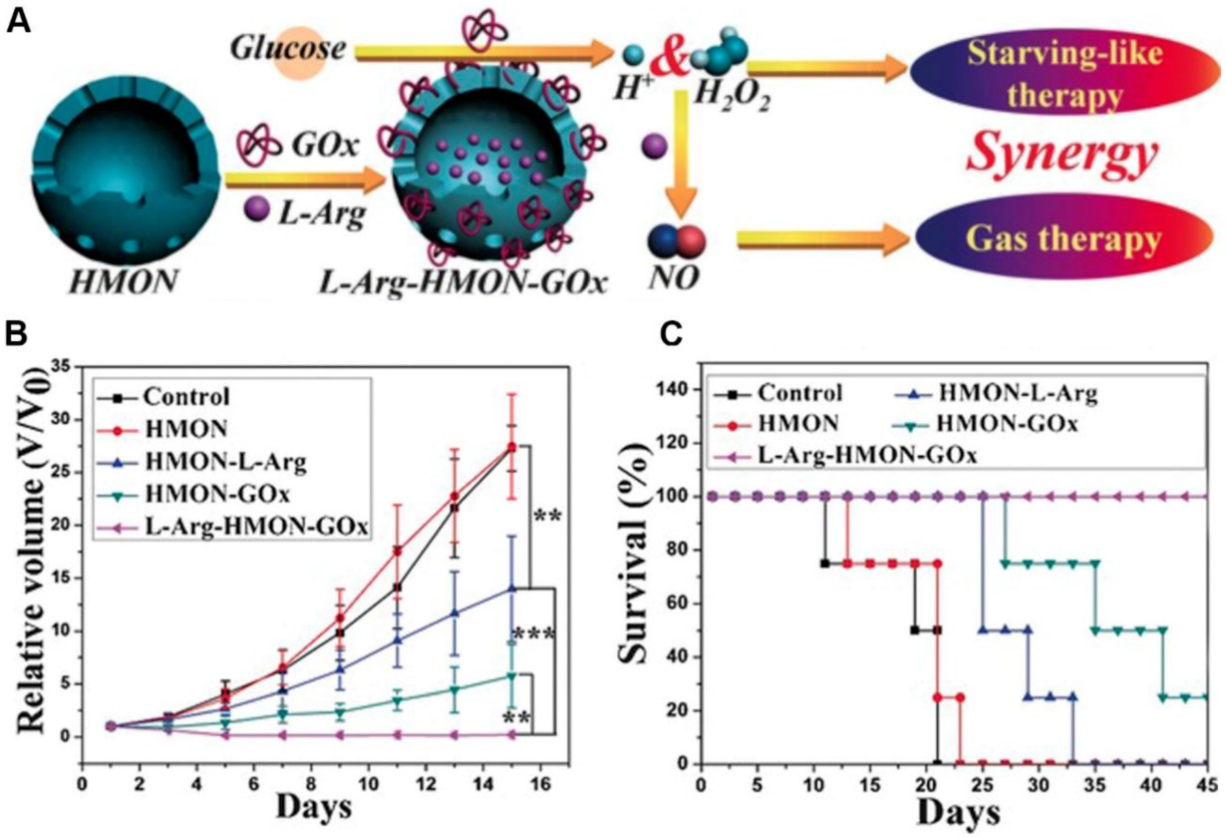
The schematic illustration of the preparation of L-Arg-HMON-GOx for cancer starvation/gas synergistic therapy (A). Tumor volume changes (B) and survival curves (C) of U87 tumor bearing mice after different treatments (*P<0.05, **P<0.01, ***P<0.001). Reproduced with permission from ref. 46. Copyright 2017, Wiley-VCH.

Previous studies have demonstrated that nitric oxide (NO) could be used as a therapeutic gas for cancer therapy through the nitrosation of mitochondria and DNA or enhance the efficiency of PDT or radiation therapy^123^, ^124^. L-Arginine (L-Arg) is a natural NO donor which can release NO in the presence of inducible NO synthase enzyme (iNOS) or in the presence of H_2_O_2_ 125, 126. Given this reality, Fan *et al*. employed a hollow mesoporous organosilica nanoparticle (HMON) for GOx and L-Arginine co-delivery (L-Arg-HMON-GOx) and cancer starvation/gas therapy **(Figure 8)** 46. After accumulating at the tumor site, the intratumoral glucose was transformed into gluconic acid and H_2_O_2_ by GOx. The generated H_2_O_2_ not only killed the tumor cells directly, but also enhanced the gas therapy effect through oxidizing L-Arginine into NO in the acidic TME. The *in vivo* experimental results indicated that L-Arg-HMON-GOx treated U87MG tumor bearing mice have the best tumor ablation outcome and much longer survival rate than the control groups, indicating the significantly promoted synergistic starvation/gas therapy effects.

#### 2.4.7 GOx-mediated starvation/immunotherapy

**Figure 9:**
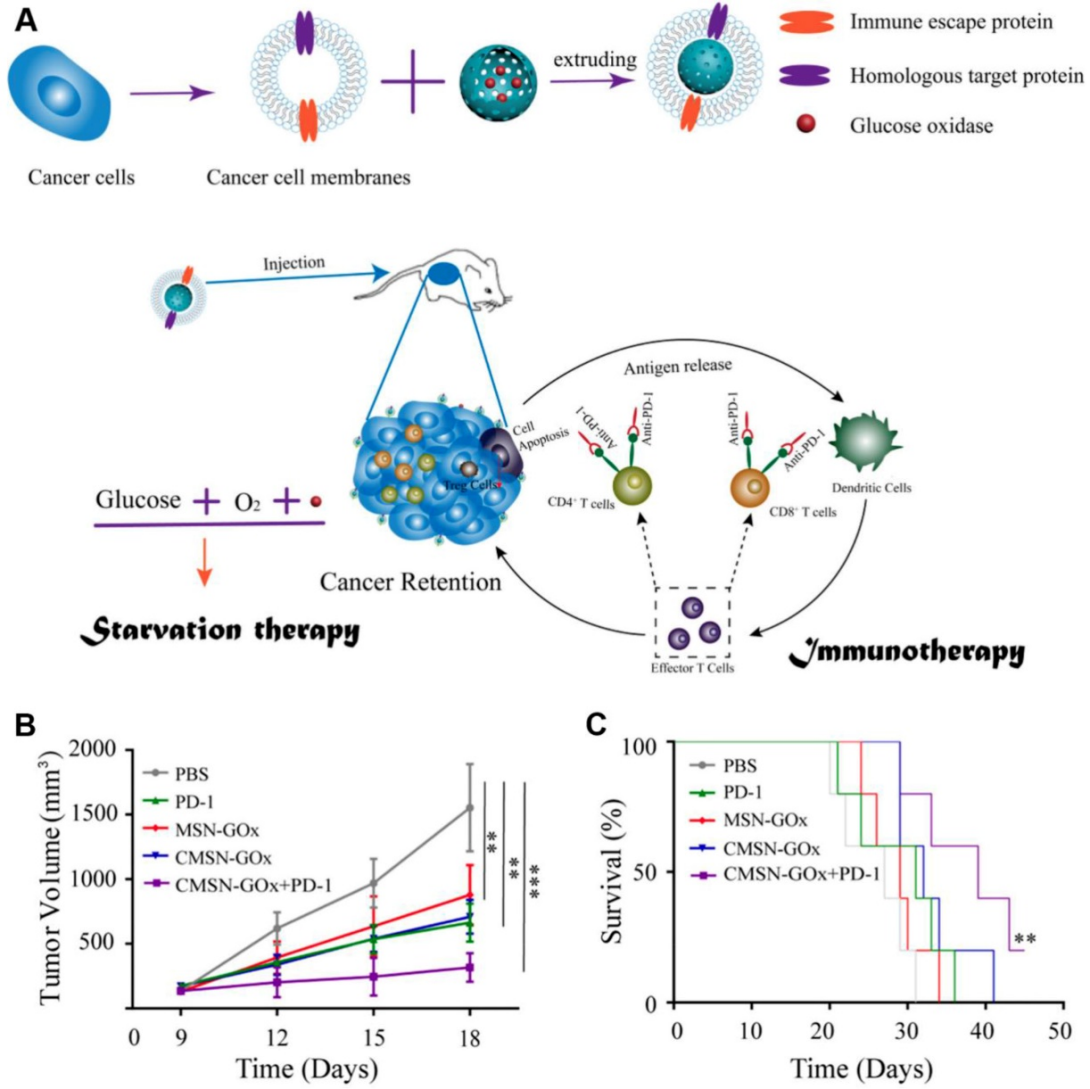
Schematic illustration of CMSN-GOx induced cancer starvation/immunotherapy (A). Tumor volume changes (n=5). (B). and survival curves (n=5). (C). Of B16F10 tumor bearing mice after different treatments. All data points are presented as mean ± standard deviation (s.d.). (*P<0.05, **P<0.01, ***P<0.001). Reproduced with permission from ref. 47. Copyright 2019, American Chemical Society.

Although cancer immune checkpoint blockade (ICB) therapy has been witnessed exciting progress in treating many types of cancers in clinic, several remaining challenges still need to be overcome in ICB-related cancer immunotherapy, such as low immune response efficacy, off-target side effects, and immune suppressive factors in TME 127-131. Combination of cancer immunotherapy with other anticancer methods has been considered as an efficient strategy for addressing these issues 132-138. For example, Xie *et al*. presented a therapeutic method combing with cancer cell membrane coated GOx-loaded mesoporous silica nanoparticles (CMSN-GOx) and anti-programmed cell death protein 1 (anti-PD-1) for cancer starvation/immunotherapy **(Figure 9)** 47. Contributing to the CM coating, CMSN-GOx was efficiently delivered to the tumor site. The released GOx could not only catalyze the glucose depletion to inhibit the tumor growth, but also induce more dendritic cells (DCs) maturation which further enhanced the antitumor efficacy of anti-PD-1.* In vivo* experimental results indicated that CMSN-GOx plus anti-PD-1 combination treatment provided more effective tumor suppression than any single therapies.

#### 2.4.8 GOx-involved multimodal synergistic therapy

As previously reported, H_2_O_2_ generated in the GOx-induced glucose oxidation could split into high toxic ∙OH radicals through Fenton reaction in the presence of Fe_3_O_4_^106^. The rising tumor temperature could further elevate the conversion efficiency of local H_2_O_2_ to ∙OH as well as enhance the antitumor ablation 71. Given this fact, Feng *et al*. developed a Fe_3_O_4_/GOx co-loaded polypyrrole (PPy)-based composite nanocatalyst (Fe_3_O_4_@PPy@GOx NC) for multimodal cancer therapy. Fe_3_O_4_@PPy@GOx NCs could selectively accumulate at the tumor site (4T1) *via* EPR effect. Thereafter, the released GOx-mediated intratumoral glucose oxidation elevated the H_2_O_2_ level and acidity of TME, which sequentially resulted in local ∙OH accumulation and tumor cell death. At the same time, the polypyrrole (PPy) component which owned a high photothermal-conversion efficiency (66.4% in NIR-II biowindow) considerably increased the tumor temperature in both in NIR-I and NIR-II biowindows, which accelerated the H_2_O_2_ disproportionation as well as enhanced the photothermal-enhanced cancer starvation/oxidation therapy.

### 2.5 Other strategies for cancer starvation therapy

Recently, types of special strategies in this field, which aimed at some critical nutrients, such as lactate and cholesterol, were also developed 39, 40, 139-141.

**Figure 10:**
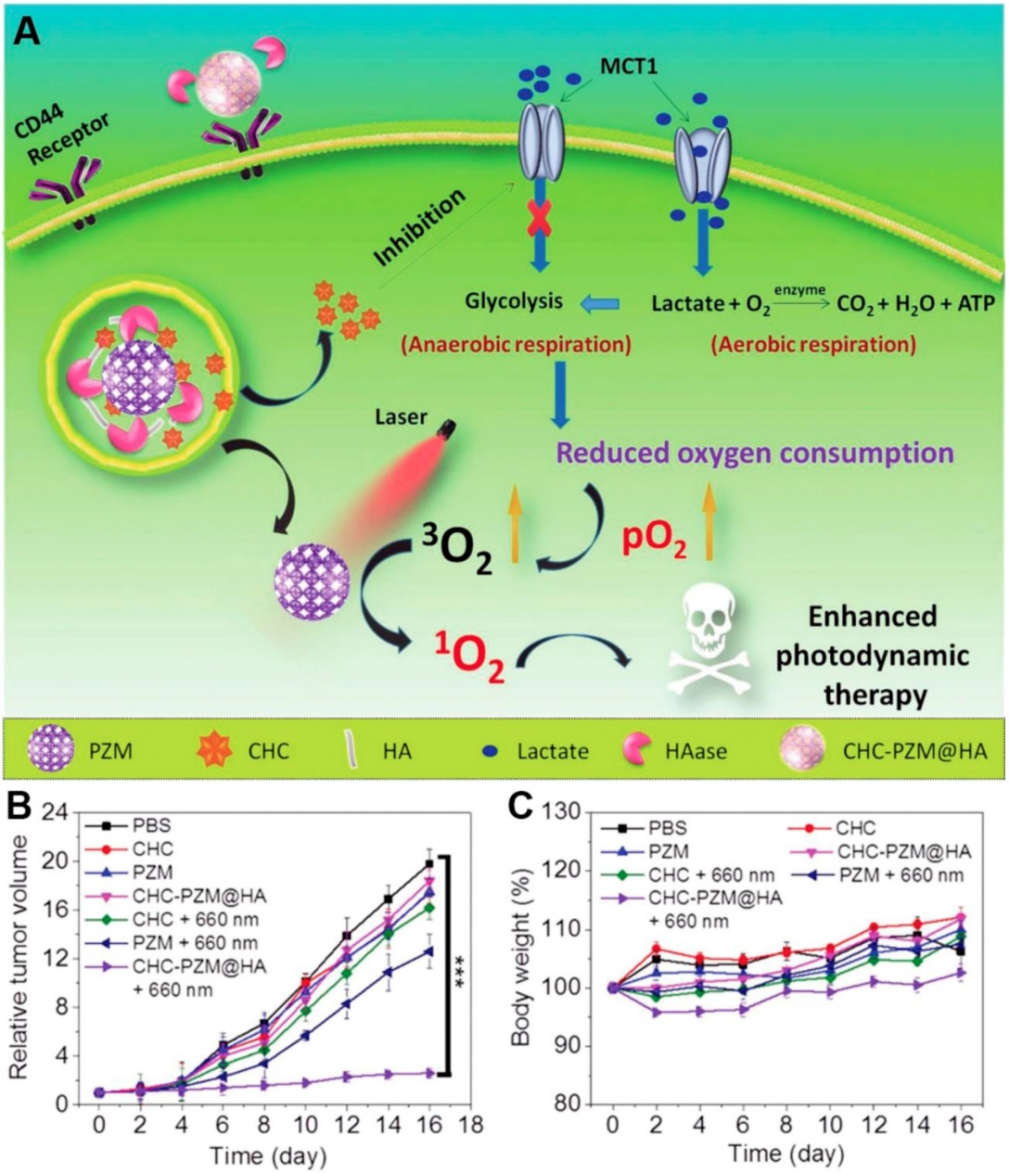
Schematic illustration of porphyrinic MOF nanoplatform mediated suppressing lactate-fueled respiration for enhanced PDT therapy (A). Tumor volume changes (n=5). (B). and body weight curves (n=5). (C). Of CT26 tumor bearing mice after different treatments. All data points are presented as mean ± standard deviation (s.d.). (***P<0.001). Reproduced with permission from ref. 40. Copyright 2018, Wiley-VCH.

Lactate, which was once considered to be the waste product of glycolysis, has been demonstrated that can “fuel” the oxidative tumor cells growth as an energy substrate 12, 142. Investigation indicated that interfering the lactate-fueled respiration could selectively kill the hypoxic tumor cells *via* inhibiting the expression of lactate-proton symporter, monocarboxylate transporter 1 (MCT1) 143. Meanwhile, the reduction of lactate uptake by inhibiting the expression of MCT1 could transform the lactate-fueled aerobic respiration to anaerobic glycolysis as well as lower the O_2_ consumption in tumor cells which would facilitate the O_2_-depleting cancer therapy. For example, Zhang and coworkers developed an *α*-cyano-4-hydroxycinnamate (CHC) loaded porous Zr (IV)-based porphyrinic metal-organic framework (PZM) NPs with HA coating for cancer combination therapy **(Figure 10)** 40. After effectively accumulating at the CT26 tumors, the released CHC could obviously decrease the expression of MCT1 and turn down the lactate uptake which leading to lower the O_2_ consumption. As a result, the PDT efficiency was markedly enhanced due to the sufficient ^3^O_2_ converting upon the laser irradiation (600 nm). Additionally, reducing the production of lactate *via* knockdown of lactate dehydrogenase A (LDHA) in tumor cells was also demonstrated that could neutralize of the tumor acidity and enhance the anti-PD-L1-mediated immunotherapy 139.

Recently, Thaxton and coworkers designed synthetic high density lipoprotein nanoparticles (HDL-NPs) with gold NPs as a size- and shape-restrictive template for lymphoma starvation therapy 39. This HDL-NPs could specially target scavenger receptor type B-1 (SR-B1), which is a high-affinity HDL receptor expressed by lymphoma cells. This combination of SR-B1 promoted the cellular cholesterol efflux and limited the cholesterol delivery, which selectively induced cholesterol starvation and cell apoptosis. The B-cell lymphoma growth was obviously inhibited after HDL-NPs treatment of B-cell lymphoma bearing mice. Furthermore, this HDL-NPs could reduce the activity of myeloid-derived suppressor cells (MDSCs), a type of innate immune cells that potently inhibit T cells, through specifically binding with SR-B1of MDSCs 140. In Lewis lung carcinoma mice model, the *in vivo* data showed that the suppression of MDSCs by HDL-NPs markedly increased CD8^+^ T cells and reduced Treg cells in the metastatic TME. After treating with HDL-NPs, the tumor growth and metastatic tumor burden were obviously reduced and the survival rate was clearly improved due to enhanced adaptive immunity.

## 3. Conclusion and outlook

As an attractive strategy for cancer treatment, nanomedicine-mediated cancer starvation therapy could selectively deprive the nutrients and oxygen supply through antiangiogenesis treatment, tumor vascular disrupting or blockade, direct depletion of the intratumoral glucose and oxygen, and other processes. Moreover, by combining with chemotherapeutic drugs, therapeutic genes, enzymes, metal NPs, hypoxia-activated prodrugs, inorganic NPs, Fenton-reaction catalysts, photosensitizers, or photothermal agents, two or more therapeutic agents could be readily integrated into one single formulation, leading to enhanced treatment outcomes (**Table 1**).

However, most innovations in this field are still in their infancy, with underlying challenges regarding clinical translation that need to be assessed in detail. For example, the biosafety of these nanomaterials is still significantly concerned, especially for the non-biodegradable formulations. Although the biosafety assessment of these materials could be systematically evaluated through animal models, long-term internal metabolic behaviors and related toxicity should be thoroughly investigated before clinical application. Another major concern is the aggravating hypoxia level that may accelerate the tumor invasion and metastasis in the progress of tumor starvation therapy. Detailed studies should be performed to confirm whether cancer starvation therapy could turn on the tumor metastasis switch by elevating the hypoxic TME, which would also help to develop new combination strategies for offering synergistic effects. Moreover, in addition to elevating the hypoxia level, these cancer starvation-based methods could also increase the intratumoral acidity and/or promoting the intracellular oxidative stress. It remains unknown how these changes influence the local and systemic immune responses. The advances in cancer immunotherapy would offer new insights and perspectives for further evolving cancer starvation-based treatments 144.

## 4. Abbreviations

## Figures and Tables

**Table 1:** Representative formulations for nanomedicine-mediated cancer starvation therapy described in this review.

Strategies	Materials	Therapeutics	Administration route	Treatments	Tumor models	Ref.
Antiangiogenesis-related cancer starvation therapy	*α*_v_*β*_3_-integrin targeted perfluorocarbon NPs	Docetaxel-prodrug	*i.v.* injection	Antiangiogenesis therapy	Vx2 tumor bearing rabbits	54
	Chitosan NPs	Ursolic acid	Oral administration	Antiangiogenesis therapy	H22 tumor bearing mice	145
	PGA-PTX-E-[c(RGDfK)_2_]	PTX	*i.v.* injection	Antiangiogenesis therapy	4T1 tumor bearing mice	57
	Recombinant human endostatin conjugated AuNPs	Recombinant human endostatin	Subcutaneous injection	Antiangiogenesis therapy	SW620 tumor bearing mice	146
	MSNs	Tanshinone IIA	*i.v.* injection	Antiangiogenesis therapy	HT-29 tumor bearing mice	53
	M-MSN@PEI-PEG-KALA NPs	anti-VEGF siRNA	*i.v.* injection	Antiangiogenic gene therapy	A549 tumor bearing mice	43
	*α*_v_*β*_3_-integrin-targeted lipid-encapsulated NPs	Fumagillin prodrug and zoledronic acid	*i.v.* injection	Dual antiangiogenic therapy	Vx2 tumor bearing rabbits	55
	Thiolated-glycol chitosan formed NPs	Bevacizumab and poly- anti-VEGF siRNA	*i.v.* injection	Dual antiangiogenic therapy	A431 tumor bearing mice	65
	Polymer-lipid hybrid NPs	DOX and MMC	*i.v.* injection	Antiangiogenesis therapy and chemotherapy	MDA-MB 435/LCC6/WT tumor bearing mice and MDA-MB 435/LCC6/MDR1 tumor bearing mice	34
	pH and thermo-responsive MSNs	DOX and MTX	*i.v.* or oral administration	Antiangiogenesis therapy and chemotherapy	OSCC tumor bearing mice	62
	PEGylated lipid bilayer-supported MSNs	AXT and CST	*i.v.* injection	Antiangiogenesis therapy and chemotherapy	SCC7 tumor bearing mice	147
	MMP-2 responsive mPEG-peptide diblock copolymer (PPDC) NPs	CPT and Sorafenib	*i.v.* injection every	Antiangiogenesis therapy and chemotherapy	HT-29 tumor bearing mice	63
	pH-sensitive poly(beta-amino ester) copolymer NPs	DOX and Cur	*i.v.* injection	Antiangiogenesis therapy and chemotherapy	SMMC 7721 tumor bearing mice	64
	Captopril-polyethyleneimine conjugated AuNPs	Captopril and siRNA	*i.v.* injection	Antiangiogenesis and gene therapy	MDA-MB-435 tumor bearing mice	66
	Calcium phosphate NPs	anti-VEGF shRNA and yCDglyTK	Intraperitoneal or intratumoral administration	Antiangiogenic gene therapy and suicide gene therapy	SGC7901 tumor bearing mice	67
	CTX-coupled SNALP-formulated anti-miR-21 oligonucleotides	CTX and anti-miR-21	*i.v.* or oral administration	Antiangiogenesis therapy and gene therapy	GBM tumor bearing mice	68
	RhoJ antibody modified Au@I NPs	RhoJ antibody	*i.v.* injection	Antiangiogenesis therapy and radiotherapy	Patient-derived tumor xenografts	61
	AuNPs	anti-VEGF siRNA	Intratumoral injection and laser irritation (655 nm)	Antiangiogenic gene therapy and photothermal therapy	PC-3 tumor bearing mice	31
	Near infrared probe iron oxide NPs	Bevacizumab	*i.v.* injection	Antiangiogenesis therapy and imaging	4T1 tumor bearing mice	58
	Sorafenib and Ce6 formed carrier-free NPs	Sorafenib and Ce6	*i.v.* injection and laser irritation (660 nm)	Antiangiogenesis therapy and photodynamic therapy	HSC3 tumor bearing mice	72
VDAs-based cancer starvation therapy						
	DOX-PLGA-conjugate NPs	Free CA4 and DOX	*i.v.* injection	Starvation therapy and chemotherapy	Lewis lung carcinoma xenografts and B16F10 tumor bearing mice	23
	LyP-1 coated liposomes	Free Ombrabulin and DOX	*i.v.* injection	Starvation therapy and chemotherapy	MDA-MB-435 tumor bearing mice	74
	Poly(L-glutamic acid)-*g*-mPEG NPs	Free CA4P and CDDP	*i.v.* injection	Starvation therapy and chemotherapy	MDA-MB-435 tumor bearing mice	75
	A15-PGA-CDDP NPs	Free DMXAA and CDDP	*i.v.* injection	Starvation therapy and chemotherapy	C26 tumor bearing mice	42
	CA4-NPs	CA4	*i.v.* injection	Starvation therapy	C26 tumor bearing mice	28
	mPEG-*b*-PHEA-DMXAA conjugate NPs	DMXAA and DOX	*i.v.* injection	Starvation therapy and chemotherapy	MCF-7 tumor bearing mice	76
	CA4-NPs	CA4 and TPZ	*i.v.* injection	Starvation therapy and chemotherapy	4T1 tumor bearing mice	27
	CA4-NPs and MMP9-DOX-NPs	CA4 and DOX	*i.v.* injection	Starvation therapy and chemotherapy	4T1 and C26 tumor bearing mice	78
Vascular blockade-induced cancer starvation therapy	CREKA modified IONPs and CRKDKC modified iron oxide nanoworms	Two tumor-homing peptide of CREKA and CRKDKC	*i.v.* injection	Starvation therapy	22Rv1 tumor bearing mice	88
	DNA nanorobots	Thrombin	*i.v.* injection	Starvation therapy	MDA-MB-231 tumor bearing mice	26
	PVP-modified Mg_2_Si NPs	Mg_2_Si	Intratumoral injection	Starvation therapy	4T1 tumor bearing mice	11
	TPZ-MNPs	Mg_2_Si and TPZ	Intratumoral injection	Starvation therapy and hypoxia-activated chemotherapy	4T1 tumor bearing mice	38
GOx-mediated cancer starvation therapy	GOx-MnO_2_@HA NPs	GOx and MnO_2_	Intratumoral injection	Starvation therapy	CT-26 tumor bearing mice	98
	Large pore-sized dendritic silica NPs	GOx and Fe_3_O_4_ NPs	Intratumoral or *i.v.* injection	Starvation therapy and oxidation therapy	4T1 and U87 tumor xenografts	106
	PEG-*b*-P(PBEM-*co*-PEM) NPs	GOx and QM	*i.v.* injection	Starvation therapy and oxidation therapy	A549 tumor bearing mice	101
	Poly(FBMA-*co*-OEGMA) nanogels	GOx	Intratumoral injection	Starvation therapy and oxidation therapy	C8161 tumor bearing mice	96
	Fe_5_C_2_-GOx@MnO_2_ NCs	GOx, Fe_5_C_2_ and MnO_2_	*i.v.* injection	Starvation therapy and oxidation therapy	U14 and 4T1 tumor bearing mice	107
	GOx-CPO@ZIF-8@NM NPs	GOx and CPO	*i.v.* injection	Starvation therapy and oxidation therapy	4T1 tumor bearing mice	109
	ATP-responsive GOx@ZIF@MPN NPs	GOx and Fe(III)/Fe(II)	Intratumoral injection e	Starvation therapy and oxidation therapy	4T1 tumor bearing mice	108
	FDMSNs@GOx@HA	GOx and Ferrocene	*i.v.* injection	Starvation therapy and oxidation therapy	HeLa tumor bearing mouse	148
	GOx@PCPT-NR	GOx and CPT prodrug	*i.v.* injection	Starvation therapy and chemotherapy	A549 tumor bearing mice	41
	Mem@GOx@ZIF-8@BDOX) NPs	GOx and BDOX	*i.v.* injection	Starvation therapy and chemotherapy	4T1 tumor bearing mice	99
	MSNs-GOx/PLL/HA	GOx and PTX	*i.v.* injection	Starvation therapy and chemotherapy	HepG2 tumor bearing mice	149
	Yolk-shell tetrasulfide bond bridged dendritic MONs	GOx and AQ4N	Intratumoral injection o	Starvation therapy and hypoxia-activated chemotherapy	4T1 tumor bearing mice	33
	TGZ@eM NRs	GOx and TPZ	*i.v.* injection	Starvation therapy and hypoxia-activated chemotherapy	CT26 tumor bearing mice	13
	HA-coated CaCO_3_ NPs	GOx and TPZ	*i.v.* injection	Starvation therapy and hypoxia-activated chemotherapy	CT26 tumor bearing mice	100
	Liposomes	GOx and AQ4N	Intratumoral injection	Starvation therapy and hypoxia-activated chemotherapy	4T1 tumor bearing mice	30
	BCE_TPZ_@(GOx+CAT)	GOx, CAT and TPZ	*i.v.* injection	Starvation therapy and hypoxia-activated chemotherapy	EMT-6 tumor bearing mice	94
	GOx conjugated polymer dots	GOx	Intratumoral injection and laser irritation (460 nm)	Starvation therapy and photodynamic therapy	MCF-7 tumor bearing mice	115
	Mem@catalase@GOx@PCN-224 bioreactor	GOx, CAT and TCPP	*i.v.* injection and laser irritation (660 nm)	Starvation therapy and photodynamic therapy	4T1 tumor bearing mice	44
	HMSNs	GOx and Ce6	*i.v.* injection	Starvation therapy and photodynamic therapy	B16F10 metastatic tumor bearing mice	32
	Hollow-MnO_2_-GOx-Ce6@CM	GOx, MnO_2_ and Ce6	*i.v.* injection and laser irritation (655 nm)	Starvation therapy and photodynamic therapy	B16F10 tumor bearing mice	150
	rMGB	GOx, MnO_2_ and Ce6	*i.v.* injection and laser irridation (660 nm)	Starvation therapy and photodynamic therapy	4T1 cancer bearing mice	120
	PEGylate HA-functionalized PHPBNs	GOx	*i.v.* injection and NIR laser irritation (808 nm)	Starvation therapy and photothermal therapy	HepG2 tumor bearing mice	121
	BSA-directed two-dimensional MnO_2_ nanosheet	MnO_2_	*i.v.* injection and NIR laser irritation (808 nm)	Starvation therapy and photothermal therapy	U87MG tumor bearing mice	122
	GOx-conjugated silver nanocubes	GOx and Ag ions	Intratumoral injection	Starvation therapy and metal ion therapy	4T1 tumor bearing mice	113
	HMONs	L-Arg and GOx	Intratumoral injection	Starvation therapy and gas therapy	U87MG tumor bearing mice	46
	CMSNs	GOx and anti-PD-1	*i.v.* injection	Starvation therapy and immunotherapy	B16F10 tumor bearing mice	47
	Fe_3_O_4_@PPy@GOx NCs.	GOx, Fe_3_O_4_ NPs and PPy	*i.v.* injection and NIR laser irradiation (808 or 1064 nm)	Starvation therapy, oxidation therapy and photodynamic therapy	4T1 tumor bearing mice	45
	CPT@MOF(Fe)-GOx	GOx, Fe^3+^ and CPT	Intratumoral injection	Starvation therapy, oxidation therapy and chemotherapy	HeLa tumor bearing mice	151
	MGH nanoamplifier	GOx and MIL-100	*i.v.* injection, NIR laser irradiation (808 nm)	Starvation therapy, oxidation therapy, photothermal therapy and imaging	4T1 tumor bearing mice	110
Other strategies for cancer starvation therapy	HDL-AuNPs	HDL	*i.v.* injection	Starvation therapy	B-cell lymphoma xenografts	39
	HDL-AuNPs	HDL	*i.v.* injection	Starvation therapy and immunotherapy	LLC tumor bearing mice and melanoma metastatic lung colonization mice model	140
	CHC-PZM@HA	CHC and PZM	*i.v.* injection, laser irradiation (660 nm)	Starvation therapy and photodynamic therapy	CT26 tumor bearing mice	40
	Mn-D@BPFe-A NPs	DOX, Fe^3+^		Starvation therapy, chemotherapy and photodynamic therapy	HepG2 tumor bearing mice	141

**Table 2:** List of abbreviations

∙OH	Hydroxyl radical
^1^O_2_	Singlet oxygen
2D	Two-dimensional
4T1	Mice breast tumor cell line
22Rv1	Human prostate cancer cell line
A431	Human epidermoid carcinoma cell line
A549	Human lung cancer cell line
Ag	Silver
AIA	Angiogenesis inhibiting agent
anti-PD-1	Programmed cell death protein 1 antibody
anti-PD-L1	Programmed cell death ligand 1 antibody
AQ4N	Banoxantrone dihydrochloride
AXT	Axitinib
ATP	Adenosine triphosphate
Au NP	Gold nanoparticle
B16F10	Mouse melanoma cell line
BDOX	H_2_O_2_-sensitive doxorubicin prodrug
BPEI	Branched polyethylenimine
BSA	Bovine serum albumin
BCE	PEG-*b*-PHEMA crosslinked CPZ loaded BSA and CAT/GOx nanoclustered enzymes
C26	Murine colon cancer cell line
C8161	Human melanoma cell line
CT26	Mouse colon cancer cell line
CA4	Combretastatin A4
CA4P	Combretastatin A4 disodium phosphate
CA4-NPs	Poly(L-glutamic acid)-CA4 conjugate nanoparticles
CDDP	Cisplatin
Ce6	Chlorin e6
CHC	*α*-cyano-4-hydroxycinnamate
CM	Cell membrane
CMSNs	Cancer cell membrane coated mesoporous silica nanoparticles
CPNP	Calcium phosphate nanoparticle
CPO	Chloroperoxidase
CPPO	Bis[2,4,5-trichloro-6-(pentyloxycarbonyl)phenyl]oxalate
CPT	Camptothecin
CST	Celastrol
CTX	Chlorotoxin
Cur	Curcumin
DDS	Drug delivery system
DCs	Dendritic cells
DMXAA	5,6-dimethylxanthenone-4-acetic acid
DOX	Doxorubicin
ECs	Endothelial cells
EEPT	Enzyme-enhanced phototherapy
eM	Erythrocyte membrane
EMT-6	Mouse mammary cancer cell line
EPR	Enhanced permeability and retention
FDA	Food and Drug Administration
FDMSNs	Ferrocene-functionalized dendritic mesoporous silica nanoparticles
GBM	Human glioblastoma cell line
GO	Graphene oxide
GOx	Glucose oxidase
GSH	Glutathione
H_2_O_2_	Hydrogen peroxide
HA	Hyaluronic acid
HAP	Hypoxia-activated prodrug
HDL	High density lipoprotein
HFR	Heparin-folic acid-retinoic acid conjugate
HMON	Hollow mesoporous organosilica nanoparticle
H22	Mouse hepatocellular carcinoma cell line
HepG2	Human liver cancer cell line
HSC3	Human oral squamous carcinoma cell line
HSP	Heat shock protein
HT-29	Mouse colon tumor cell line
ICG	Indocyanine green
ICB	Immune checkpoint blockade
iNGR-NP	iNGR-modified PEG-PLGA nanoparticle
iNOS	NO synthase enzyme
IONP	Iron oxide nanoparticle
*i.v.*	Intravenous injection
LyP-1	Phage-displayed cyclic peptide
LLC	Lewis lung carcinoma cell line
MCF-7	Human breast cancer cell line
MCT1	Monocarboxylate transporter 1
MDA-MB-435	Human breast cancer cell line
MDA-MB-231	Human breast cancer cell line
MDR	Multidrug resistance
MDSCs	Myeloid-derived suppressor cells
Mg_2_Si	Magnesium silicide
MGP	GOx and MnCO coloaded poly(lactic-co-glycolic acid) nanoparticles
MIL-100	Ferric ions contained metal organic framework
MGH	GOx loaded MIL-100 with polydopaminemodified hyaluronic acid coating
MMC	Mitomycin C
MMP	Matrix metalloproteinase
MnO_2_	Manganese dioxide
M-NS	BSA-directed two-dimensional MnO_2_ nanosheet
MOF	Metal-organic framework
MOST	Multispectral optoacoustic tomography
MPN	Metal polyphenol network
MSN	Mesoporous silica nanoparticle
MTX	Methotrexate
nano-DOA	Nano-deoxygenation agent
NC	Nanocatalyst
NIR	Near-infrared
NM	Neutrophil membrane
NO	Nitric oxide
NP	Nanoparticle
NR	Nanoreactor
O_2_	Oxygen
OCl^-^	Hypochlorite ion
OSCC	Oral squamous carcinoma cell line
PC-3	Human prostate cancer cell line
Pdot	Polymer dot
PDT	Photodynamic therapy
PFC	Perfluorocarbon
PLA	Poly-L-arginine
PLL	poly (L-lysine)
PLGA	Polylactic-*co*-glycolic acid
PEG	Poly(ethylene glycol)
PEG-*b*-PHEMA	poly(ethylene glycol)-*block*-poly(2-hydroxyethyl methacrylate)
PLG-*g*-mPEG	Poly(L-glutamic acid)-*g*-methoxy poly(ethylene glycol)
PPy	Polypyrrole
PTT	Photothermal therapy
PTX	Paclitaxel
PVP	Polyvinyl pyrrolidone
PZM	Porous Zr (IV)-based porphyrinic metal-organic framework
RA	Retinoic acid
RBC	Red blood cell
RGD	Arginine-glycine-aspartic acid
rMGB	Biomimetic hybrid nanozyme with a GOx/MnO_2_/BSA-Ce6 core and red blood cell membrane coating
ROS	Reactive oxygen species
SCC7	Murine squamous cell carcinoma cell line
SGC7901	Gastric cancer cell line
shVEGF	VEGF-targeted small hairpin RNA
SiO_2_	Silicon dioxide
siRNA	Small interfering RNA
SMMC 7721	Human liver tumor cell line
SR-B1	Scavenger receptor type B-1
SW620	Human Caucasian colon adenocarcinoma cell line
TACE	Transarterial chemoembolization
TAF	Tumor angiogenesis factor
TCPP	Tetrakis (4-carboxyphenyl) porphyrin
THP	Tumor-homing peptide
TME	Tumor microenvironment
TPZ	Tirapazamine
UV	Ultraviolet
VDA	Vascular disrupting agent
VEGF	Vascular endothelial growth factor
U14	Mouse cervical subcutaneous cancer cell line
U87	Human glioblastoma cell line
U87MG	Human glioblastoma cell line
Vx2	Rabbit carcinoma cell line
yCDglyTK	Fusion suicide gene
ZIF-8	Zeolitic imidazolate framework-8
*α*_v_*β*_3_-Dxtl-PD NP	*α*_v_*β*_3_-integrin targeted perfluorocarbon nanoparticle
